# Ovarian tumours of different histologic type and clinical stage induce similar changes in lipid metabolism

**DOI:** 10.1038/s41416-018-0270-z

**Published:** 2018-10-08

**Authors:** Riikka Johanna Niemi, Elena Ioana Braicu, Hagen Kulbe, Kaisa Maria Koistinen, Jalid Sehouli, Ulla Puistola, Johanna Unelma Mäenpää, Mika Hilvo

**Affiliations:** 1 Department of Obstetrics and Gynaecology, Tampere University Hospital, Tampere, Finland; 2 Charité—Universitätsmedizin Berlin, Corporate Member of Freie Universität Berlin Humboldt-Universität zu Berlin, and Department of Gynaecology, Berlin Institute of Health, Berlin, Germany; 3 Zora Biosciences Oy, Espoo, Finland; 4 Department of Obstetrics and Gynaecology, PEDEGO Research Unit, Medical Research Center Oulu, University of Oulu and University Hospital of Oulu, Oulu, Finland; 5 Faculty of Medicine and Life Sciences, University of Tampere, Tampere, Finland

**Keywords:** Ovarian cancer, Diagnostic markers, Lipidomics

## Abstract

**Background:**

Previous results obtained from serum samples of late-stage, high-grade serous ovarian carcinoma patients showed large alterations in lipid metabolism. To validate and extend the results, we studied lipidomic changes in early-stage ovarian tumours. In addition to serous ovarian cancer, we investigated whether these changes occur in mucinous and endometrioid histological subtypes as well.

**Methods:**

Altogether, 354 serum or plasma samples were collected from three centres, one from Germany and two from Finland. We performed lipidomic analysis of samples from patients with malignant (*N* = 138) or borderline (*N* = 25) ovarian tumours, and 191 controls with benign pathology. These results were compared to previously published data.

**Results:**

We found 39 lipids that showed consistent alteration both in early- and late-stage ovarian cancer patients as well as in pre- and postmenopausal women. Most of these changes were already significant at an early stage and progressed with increasing stage. Furthermore, 23 lipids showed similar alterations in all investigated histological subtypes.

**Conclusions:**

Changes in lipid metabolism due to ovarian cancer occur in early-stage disease but intensify with increasing stage. These changes occur also in other histological subtypes besides high-grade serous carcinoma. Understanding lipid metabolism in ovarian cancer may lead to new therapeutic and diagnostic alternatives.

## Background

Prognosis of ovarian cancer improves remarkably if the disease is diagnosed at an early stage, as early detection affords better opportunities for curative treatment. Current diagnostic methods primarily include vaginal ultrasound combined with the blood test to measure cancer antigen 125 (CA 125) levels. These methods lack specificity and sensitivity, especially in non-advanced ovarian cancer.^1^ Therefore, there is a demand for new detection methods and biomarkers for distinguishing benign and borderline ovarian tumours, as well as early-stage and advanced ovarian cancer.

Malignant tumours, including ovarian cancer, adopt many metabolic abnormalities to meet the increased energy demand associated with increased cellular proliferation and tumour growth.^2^ In ovarian cancer, the metabolic alterations in tissues and body fluids have been investigated by metabolic profiling to identify biomarkers for early detection and reliable prognosis.^3–5^ Recently, using liquid chromatography-mass spectrometry (LC-MS), Gaul et al.^6^ found from serum 16 diagnostic metabolites, including many lipids and fatty acids, that distinguish early-stage ovarian cancer samples from healthy control samples. In a lipidomic study, Buas et al.^7^ showed 34 significantly altered metabolites between serous ovarian carcinoma and benign serous ovarian tumour patients, and the plasma levels of the lipids were reduced in patients with a malignant disease. Recently, our metabolomic analyses of tumour and blood samples from high-grade serous ovarian carcinoma (HGSOC) patients showed elevated concentrations of hydroxybutyric acids, implicating that these molecules could act as diagnostic and prognostic biomarkers.^8^ Subsequently, lipidomic profiling of the same samples showed an overall reduction in the levels of most of the lipid species but elevations in specific ceramide (Cer) and triacylglycerol (TAG) lipids in metastatic ovarian cancer patients.^9^

Despite several studies showing lipidomic alterations in ovarian cancer, we are not aware of any studies that confirm which lipid species are the most consistently altered. To this end, as well as to validate our published lipidomic results and extend the analyses to low malignant potential (borderline) ovarian tumours and early-stage ovarian cancers, we applied the same previously used methodology^9^ to analyse blood samples from patients with early-stage ovarian cancers. These results were subsequently compared to the results obtained from patients with benign gynaecological disease. Our further aim was to investigate whether the lipidomic alterations found in patients with HGSOC can be applied to other histological subtypes, i.e., to mucinous and endometrioid ovarian carcinoma.

## Materials and methods

### Patients and samples

We performed lipidomic profiling on two study cohorts, one from Charitè (*N* = 189) and another from Finland (*N* = 165, from Tampere (*N* = 111) and Oulu (*N* = 54) University Hospitals). In addition, we used data from an independent, previously published study^9^ referred herein as the Charité discovery (*N* = 250). The Charité discovery study included 5 additional samples from patients with endometrioid tumours that were excluded from the original publication.^9^ Clinical characteristics of these three study cohorts are shown in Table 1. The samples from both Charité studies were serum samples, while the Finnish samples were a mixture of serum and plasma, as shown in Table 1. All samples were collected preoperatively. In total, in these three studies, 290 samples were collected from patients with malignant ovarian tumours, 25 samples from subjects with borderline ovarian tumours and 289 from women with benign gynaecological tumours, endometriosis, infection or other conditions. The diagnosis of invasive and borderline ovarian tumours was based on the World Health Organization Classification.^10^ The gynae-pathologists at the respective hospitals (University Hospitals of Oulu and Tampere, Finland and Charité, Berlin, Germany) did the histological analyses, and immunohistochemistry was used when needed. The Charité samples were collected at the Tumor Bank—Ovarian Cancer Network (www.toc-network.de) at the Charitè Medical University (Berlin, Germany) between July 2013 and September 2016. The Finnish samples, from Tampere University Hospital and Oulu University Hospital, were collected between February 2011 and November 2014 and between January 2009 and December 2015, respectively.

**Table 1:** Clinical characteristics of the study cohorts

	Charité	Finland	Charité discovery
*Malignant*	62	76	152
Age	57 (50–72)***	58 (51–64)*	59 (50–67)***
Histology			
Serous	41	29	147
Mucinous	6	18	
Endometrioid	9	14	5
Other	6	15	
Stage			
I & II	26	52	8
III & IV	33	22	133
NA	3	2	11
Sample			
Serum	62	22	152
Plasma		54	
*Borderline*	18	7	
Age	51 (44–57)^NS^	63 (56–67)^NS^	
Histology			
Serous	13	5	
Mucinous	2	2	
Other	3		
Stage			
I & II	12	7	
III & IV	3		
NA	3		
Sample			
Serum	18	7	
Benign	109	82	98
Age	49 (40–58)	62 (56–69)	41 (31–55)
Diagnosis			
Other	7	2	43
Uterine fibroid	7	1	25
Cyst	4	9	1
Cystic teratoma	12	8	5
Functional cyst	22		
Inclusion cyst	3		
Endometrioid cyst	5		
Non-ovarian cyst		4	
Cystadenoma	32	2	4
Mucinous cystadenoma		3	2
Cystadenofibroma	7	10	2
Serous cystadenoma		34	3
Brenner tumour	1	2	1
Fibroma/thecoma		5	
Fibroadenoma		1	
Incomplete abortion			5
Adnexitis			5
Endometriosis	9	1	2
Sample			
Serum	109	82	98

*NS* not significant

For age, the values represent median and interquartile range and *p*-values in the comparison against the control group are denoted as follows: ***p < 0.001; **p < 0.01; *p < 0.05

### Lipidomic analysis of serum samples (LC-tandem mass spectrometry)

The samples were randomised within each cohort before lipidomic analysis. The lipidomic analysis has been previously described in detail.^9^ Briefly, lipidomic analyses were performed using two platforms, a global screening method and a phosphosphingolipid platform. For the screening method, 10 µl of sample was needed for the extraction of the lipids using a modified Folch extraction.^11^ For the phosphosphingolipid method, 25 µl of sample was needed for the extraction of lipids using protein precipitation in methanol.

Lipidomic screening and phosphosphingolipid platforms were both analysed on a hybrid triple quadrupole/linear ion trap mass spectrometer (QTRAP 5500, AB Sciex, Concords, Canada) equipped with ultra-high-performance liquid chromatography (Nexera-X2, Shimadzu, Kyoto, Japan). Chromatographic separation of the lipidomic screening platform was performed on an Acquity BEH C18, 2.1 × 50 mm id. 1.7 µm column (Waters Corporation, Milford, MA, USA). Chromatographic separation of the phosphosphingolipid platform was performed on an AQUASIL C18, 2.1 × 50 mm, 5 µm (Thermo Fisher Scientific, Waltham, MA, USA) column set at 60 °C. For the MS analysis, a targeted approach in the positive ion mode was used for both platforms. The data were collected using a scheduled multiple reaction monitoring (sMRM™) algorithm for the lipidomics screening platform^12^ and MRM for phosphosphingolipids. The lipidomic data were processed using Analyst and MultiQuant 3.0 software (AB Sciex), and the area or height ratios of the analyte and its corresponding internal standard (IS) peak were normalised with the IS amount and the sample volume. The details of the chromatography and mass spectrometry conditions have been previously described.^9^

The number of lipids and the mean coefficient of variation for each lipid class, determined from the quality control samples (6 in each 96-well plate), are shown in Supplementary Table S1. The list of all analysed lipids has been published previously.^9^

### Statistical analyses

Group comparisons (patients vs. controls) were performed by calculating the mean relative difference between the groups, and the *p*-values were determined by parametric *t*-tests on log-transformed concentrations. R version 3.4.2 was used for all statistical analyses. Tableau 10.1 was used for heatmap visualisations. For diagnostic calculations, logistic regression models were developed using all samples in the Charité cohort and tested in the Finnish cohort. The area under the curve (AUC) values were determined using the *pROC* package.^13^ The top models presented in the article were selected by calculating the sum of the AUC values in both cohorts, and selecting the models with the highest values.

## Results

### Validation of altered lipidomic profile in ovarian cancer patients

To validate the lipidomic alterations detected in ovarian cancer patients, we determined which lipids were similarly altered between the patients and the controls in the two study cohorts (Charité and Finland), in addition to the previously published Charité discovery cohort (Table 1), provided that the change between the patients and the controls was significant in at least two cohorts. The results confirmed that ovarian cancer causes wide lipidomic changes as 155 lipids showed the same direction of change in all cohorts, and most of these changes were also statistically significant in all three independent cohorts (Supplementary Table S2). All further analyses were limited to these 155 lipids.

### Lipidomic changes emerge in early-stage ovarian cancer patients

**Fig. 1:**
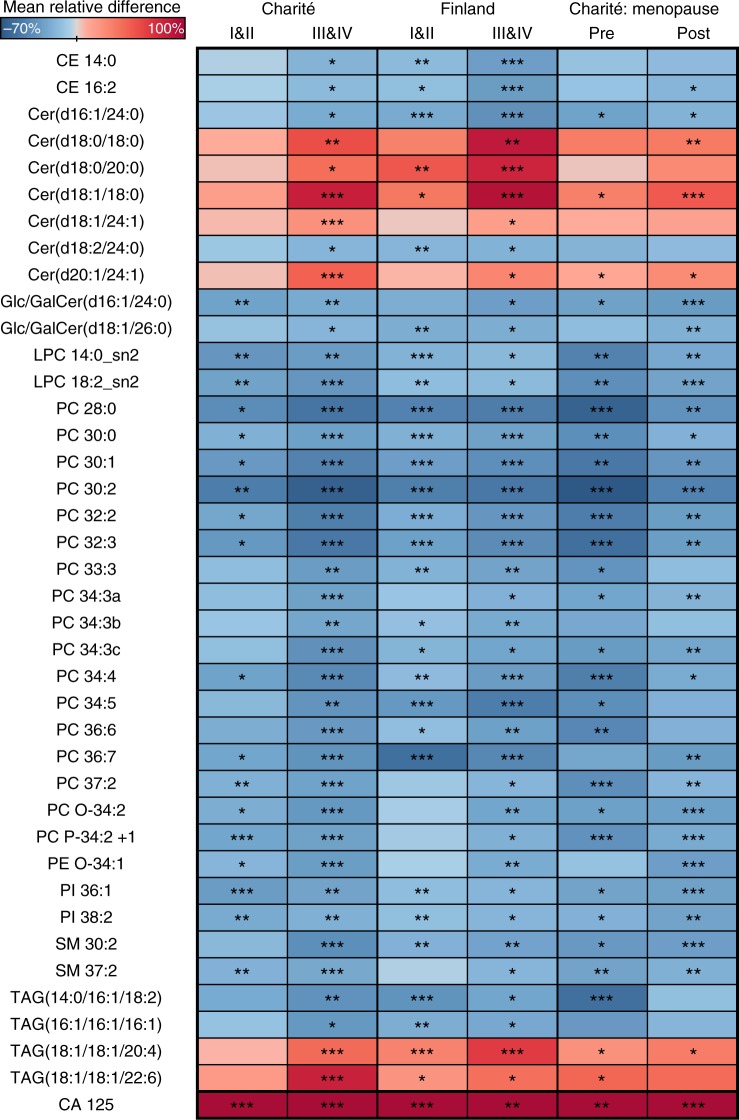
Heatmap showing lipidomic changes in early- (I/II) and late-stage (III/IV) ovarian cancer patients. In addition, the results are shown in pre- and postmenopausal patients of all stages. The difference is calculated relative to controls. The colour scale (from −70 to 100%) is adjusted according to the lipids, in cancer patients CA 125 showed mean elevation higher than 100%. ***p < 0.001; **p < 0.01; *p < 0.05. Charité study had 60 premenopausal controls and 17 cancer cases as well as 48 postmenopausal controls and 42 cancer cases

To identify which lipids have the best diagnostic potential, or those already altered in early-stage (I/II) cancer, we selected lipids that showed consistent increase or decrease both in stage I/II vs. controls and stage III/IV vs. controls, including all cohorts and histological subtypes. In addition, the lipids had to be significantly altered at least in stage III/IV patients in the Charité and Finnish cohorts. This approach resulted in 39 lipids, which are shown in a heatmap in Fig. 1. Samples from patients with ovarian cancer revealed a consistent decrease in the concentration of most of the analysed lipid classes and included phospholipids (phosphatidylcholines (PCs), lysophosphatidylcholines (LPCs), and phosphatidylinositols (PIs)), cholesteryl esters, glucosyl/galactosyl Cers, and sphingomyelins. In turn, an increase was observed in many Cers with certain fatty acyl (FA) side-chain compositions. Cers with 18:0, 20:0 and 24:1 FAs were increased, while 24:0 FA-containing Cers were decreased. The TAG lipid species also showed a variable trend depending on the FA side chains; TAGs with shorter FA side chains were decreased, whereas those with longer FA side chains were increased. In many lipid species, the alterations were more significant in advanced-stage (III/IV) patients but were already present in early-stage patients (I/II) (Fig. 1). The lipidomic changes were consistent in both pre- and postmenopausal patient populations (Fig. 1).

### Tumours of various histological subtypes induce similar lipid changes

**Fig. 2:**
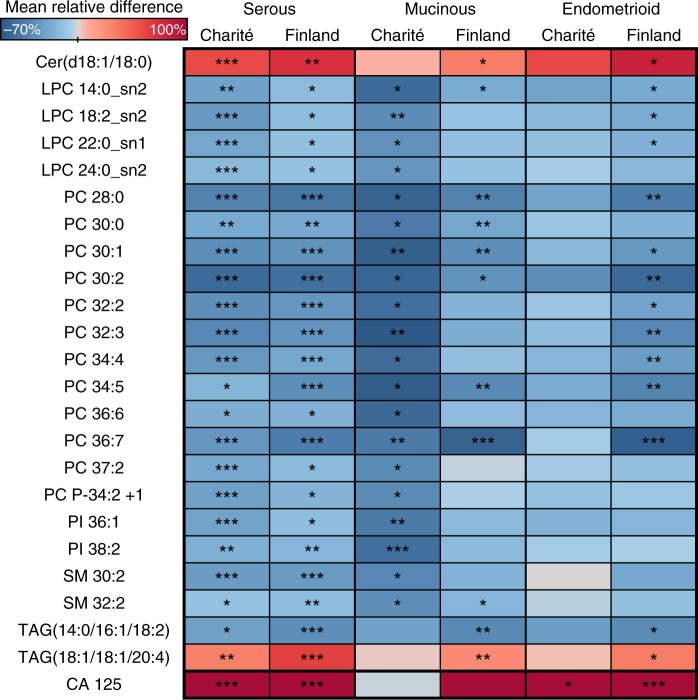
Heatmap showing lipidomic changes in ovarian cancer patients with different histological subtypes as compared to control subjects. The colour scale (from −70 to 100%) is adjusted according to the lipids, in some of the analyses CA 125 showed elevation higher than 100%. ***p < 0.001; **p < 0.01; *p < 0.05

As the previous results were derived from HGSOC patients only,^9^ we investigated whether some changes in lipid species are also significant in patients with other histological subtypes (mucinous and endometrioid). Thus, we selected lipids showing the same direction of alteration in all histological subtypes of the Charité and Finnish cohorts. In addition, the selected lipids had to be significant in either mucinous or endometrioid subtypes in either of the cohorts. Twenty-one of 23 lipids were decreased in all histological subtypes (Fig. 2), and only Cer(d18:1/18:0) and TAG(18:1/18:1/20:4) were increased. The most significant alterations were observed in PCs and LPCs. All lipid changes were significant in the serous subtype, which was expected based on the large number of cases in both cohorts. Interestingly, CA 125 was not significantly altered in mucinous subtype samples, while most lipid changes were significant in the Charité cohort despite a low number of mucinous cases (*N* = 6). For endometrioid histology, none of the lipids were significant in the Charité cohort (*N* = 9), whereas the Finnish cohort, with a slightly greater number of cases (*N* = 14), showed significant alterations.

### Fewer lipid changes are seen in borderline tumours than in malignant tumours

**Fig. 3:**
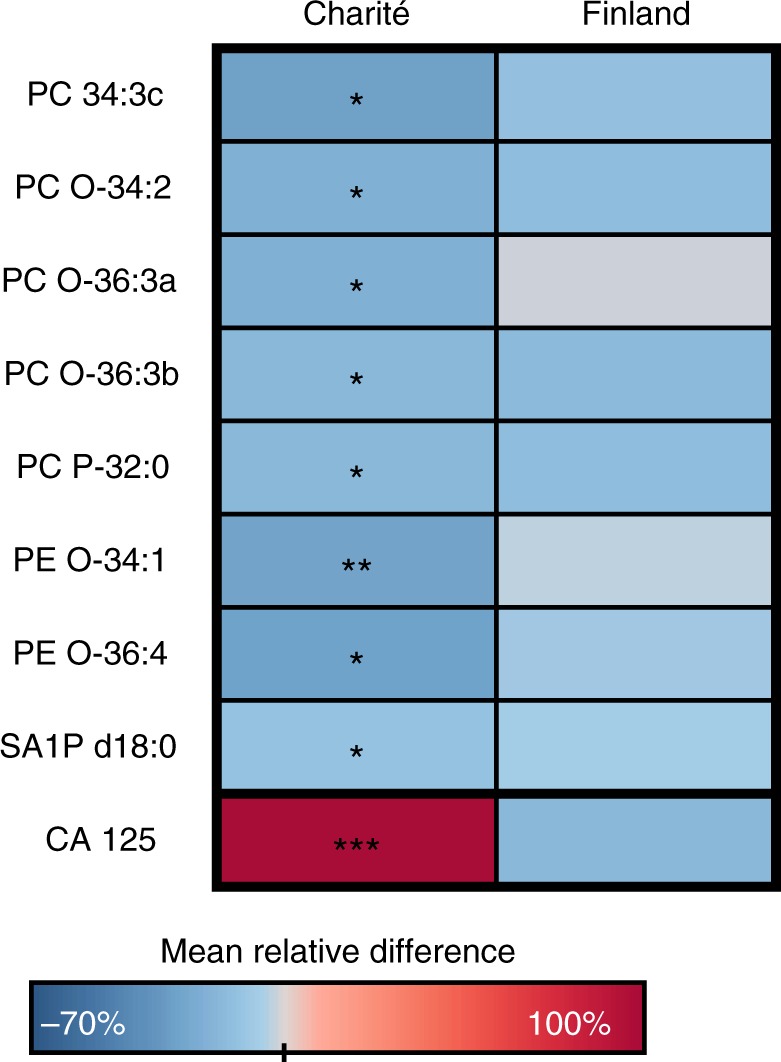
Heatmap showing lipidomic changes in patients with borderline tumours as compared to control subjects. The colour scale is adjusted according to the lipids (from −70 to 100%), CA 125 showed elevation higher than 100% in the Charité cohort. ***p < 0.001; **p < 0.01; *p < 0.05

We also analysed whether the observed lipidome alterations are present in borderline ovarian tumours. When only those lipids that were altered in the same direction in both cohorts and significant in at least one of them were selected, there were only a few significant alterations (Fig. 3). Thus, it appears that borderline tumours do not cause as much of a change to the lipidome as malignant tumours.

### Lipids improve the diagnostic value of CA 125 for the detection of early-stage cancer

Finally, we investigated whether lipids can improve the diagnostic value of CA 125. As lipid ratios have shown diagnostic value in other diseases,^14^ we investigated combinations of all lipids and lipid ratios together with CA 125. The lipids used for this analysis are shown in Fig. 1. For the ratio calculations, the increased lipids in ovarian cancer patients and CA 125 were used as numerators, and all other lipids were used as denominators. To find more robust biomarkers, those lipids and lipid ratios were excluded that were significantly different (*t*-test *p* < 0.05 and mean relative change > 10%) between control samples of the Charité and Finland cohorts. The models were generated using all subjects in the Charité cohort, and tested in the stage I/II and III/IV ovarian cancer patients separately, in addition to the validation in the Finnish cohort. As an example, the models with the highest improvement in both the Charité and Finnish cohorts are shown in Table 2. In the Charité cohort, CA 125 as a continuous variable instead of using the 35 U/mL cutoff improved the AUC values, and further improvement was seen for the detection of early-stage cases with incorporation of lipids, but not for late-stage cases where already CA 125 alone performed well. In the Finnish cohort, which had a higher proportion of other than serous malignant tumours, the AUC values for CA 125 and also the models with lipids were lower than in the Charité, but again the lipids improved the diagnostic value of CA 125 for the detection of stage I/II cancers.

**Table 2:** AUC values with 95% confidence intervals for the logistic regression models

Variable 1	Variable 2	Charité			Finland		
		All	Stage I/II	Stage III/IV	All	Stage I/II	Stage III/IV
CA 125/Glc/GalCer(d18:1/26:0)	Cer(d18:1/24:1)/LPC 14:0_sn2	0.93 (0.89–0.96)	0.87 (0.80–0.94)	0.98 (0.96–1.00)	0.76 (0.68–0.85)	0.74 (0.64–0.83)	0.93 (0.84–1.00)
Cer(d18:1/24:1)/LPC 14:0_sn2	CA 125/PC 37:2	0.93 (0.89–0.96)	0.87 (0.81–0.94)	0.98 (0.95–1.00)	0.76 (0.68–0.85)	0.73 (0.64–0.83)	0.95 (0.89–1.00)
Cer(d20:1/24:1)/LPC 14:0_sn2	CA 125/PC 37:2	0.92 (0.87–0.96)	0.85 (0.77–0.93)	0.98 (0.95–1.00)	0.77 (0.68–0.85)	0.74 (0.64–0.83)	0.95 (0.90–1.00)
Cer(d18:1/24:1)/LPC 14:0_sn2	CA 125/PI 38:2	0.92 (0.89–0.96)	0.87 (0.81–0.94)	0.97 (0.94–1.00)	0.77 (0.69–0.85)	0.75 (0.66–0.84)	0.95 (0.88–1.00)
CA 125	TAG(18:1/18:1/22:6)/LPC 14:0_sn2	0.91 (0.86–0.96)	0.83 (0.73–0.92)	0.98 (0.96–1.00)	0.78 (0.70–0.86)	0.75 (0.66–0.84)	0.89 (0.77–1.00)
TAG(18:1/18:1/22:6)/LPC 14:0_sn2	CA 125/PC 37:2	0.91 (0.86–0.96)	0.83 (0.74–0.91)	0.98 (0.96–1.00)	0.78 (0.70–0.86)	0.75 (0.66–0.84)	0.88 (0.73–1.00)
TAG(18:1/18:1/22:6)/LPC 14:0_sn2	CA 125/PC P-34:2 + 1	0.91 (0.86–0.96)	0.83 (0.75–0.92)	0.98 (0.96–1.00)	0.78 (0.70–0.86)	0.75 (0.66–0.84)	0.89 (0.75–1.00)
TAG(18:1/18:1/22:6)/LPC 14:0_sn2	CA 125/SM 37:2	0.91 (0.86–0.95)	0.83 (0.73–0.92)	0.98 (0.96–1.00)	0.78 (0.70–0.86)	0.75 (0.66–0.84)	0.91 (0.80–1.00)
Cer(d20:1/24:1)/LPC 14:0_sn2	CA 125/PI 38:2	0.91 (0.87–0.96)	0.85 (0.76–0.93)	0.97 (0.94–1.00)	0.78 (0.70–0.86)	0.76 (0.67–0.85)	0.95 (0.88–1.00)
TAG(18:1/18:1/22:6)/PC 30:0	CA 125/PC 30:0	0.90 (0.85–0.95)	0.82 (0.73–0.91)	0.98 (0.95–1.00)	0.79 (0.72–0.87)	0.77 (0.68–0.85)	0.91 (0.78–1.00)
CA 125		0.90 (0.84–0.95)	0.81 (0.71–0.90)	0.97 (0.94–1.00)	0.72 (0.62–0.81)	0.67 (0.57–0.78)	0.95 (0.91–1.00)
CA 125 (35 U/mL cutoff)		0.80 (0.73–0.86)	0.69 (0.59–0.80)	0.89 (0.84–0.94)	0.71 (0.64–0.79)	0.68 (0.60–0.76)	0.91 (0.87–0.95)

As comparison, the models are shown also for CA 125 alone or CA 125 as binary variable dichotomised by the clinically used 35 U/mL cutoff value

## Discussion

The present global lipidomics study investigating early- and advanced-stage ovarian cancer of various histological subtypes was performed to validate and extend our previous results on lipid changes in HGSOC patients. Altered lipid metabolism seems to be linked to ovarian cancer, but specific findings are still strikingly variable. Our data are in line with those earlier studies showing an overall decrease in the serum/plasma concentration of lipid metabolites^7^ and glycerophospholipids^15,16^ in ovarian cancer patients. The intensification of lipid changes in the advanced-stage ovarian cancer patients suggests that the tumours are exploiting circulating lipids and lipoproteins with proportion to their size. The overall decrease of PCs may be associated with reduction of high-density lipoprotein (HDL) cholesterol and ApoA1 in the ovarian cancer patients,^17,18^ as PCs are known to be abundant especially in the HDL particles.^19^ However, this phenomenon cannot be used to explain the increase of lipid species in ovarian cancer patients. It has been suggested that changes in lipid metabolism during ovarian cancer pathogenesis reflect higher levels of cell division,^20^ enhanced fatty acid β-oxidation,^5^ and increased cellular proliferation or motility due to increased PI3-kinase activity,^21^ yet there are likely to be additional mechanisms explaining the alterations of specific lipids.

These results confirm our previous report describing an increase in the serum concentration of Cer(d18:1/18:0), Cer(d18:0/18:0) and TAG(18:1/18:1/20:4) in ovarian cancer patients.^9^ Moreover, the phenomenon is evident at the early stages of disease development, i.e. stage I/II, but was found to become more pronounced with disease progression. In addition to HGSOC, Cer(d18:1/18:0) and TAG(18:1/18:1/20:4) were also significantly increased in mucinous and endometrioid ovarian cancer samples from the Finnish cohort. However, the number of mucinous and endometrioid carcinoma samples was likely too low in the Charitè cohort to show any significant difference. Interestingly, Cer(d18:1/18:0) and its precursor Cer(d18:0/18:0) have been associated with the development of insulin resistance and type 2 diabetes.^22–24^ Taken together, these alterations to the lipid profile and other metabolic changes, such as increase of ketone bodies,^8^ suggest that the metabolic profile of ovarian cancer patients resemble a diabetic phenotype.

Sphingolipids, especially Cers, have been linked to the development and progression of cancer,^25^ but results appear vary depending on the type of tumour.^26^ Cers are considered to have anticancer properties, to act as second messengers for cell apoptosis^25^ and to modulate cell growth.^27^ Another sphingolipid, sphingosine-1-phosphate (S1P), has opposing cellular effects to Cers.^26^ The role of sphingolipid metabolism in ovarian cancer has been investigated in a recent study in which 74 women with HGSOC were found to have significantly elevated plasma and tissue concentrations of C16-Cer, C18:1-Cer and C18-Cer compared to those of healthy controls,^28^ which is in line with our results. The researchers speculated that the increased amounts of Cers would be associated with particularly aggressive epithelial ovarian cancer cases and that the increased Cer concentrations would lead to increased conversion to S1P, as they found an elevated S1P concentration in tumour tissue. However, congruent with our data, elevation of S1P could not be observed in blood.

Buas et al.^7^ have shown reduction of all measured TAGs in the plasma of ovarian cancer patients. However, in a lipidomic analysis of low and highly aggressive ovarian cancer cell lines, TAGs increased dramatically along aggressiveness of the cells and were assumed to be the largest source of cellular energy.^29^ In a mouse model of HGSOC, compared to healthy mice, the serum levels of LPE(16:0) and PIs were decreased, while TAG(55:7) was significantly increased at early-stage cancer development.^30^ On the other hand, decreased levels of TAGs in epithelial ovarian cancer patients have been shown to predict early recurrence of cancer.^31^ In our study, only the concentrations of TAGs with longer fatty acid chains were increased or not altered, while those TAGs with short fatty acid chains were decreased. Our former study proposed that this result could be explained by genetics via low expression of the *ABCD1* gene,^9^ which is associated with transport of long-chain fatty acids into the peroxisome for β-oxidation.^32^

Phospho- and sphingolipids are the most studied lipids in regard to the pathogenesis of ovarian cancer.^33^ In 2004, it was shown that plasma levels of lysophospholipids varied between healthy controls and ovarian cancer patients, as well as pre- and postoperatively.^34^ Moreover, in a pathway analysis, glycerophospholipid (LPCs and PCs) metabolism was a main dysregulated pathway in the pathogenesis of ovarian carcinoma.^35^ Alteration of LPC levels may be caused by the binding and activation of specific cell surface G protein-coupled receptors, which can activate cell growth and proliferation.^36^ Altered LPCs and lysophosphatidylethanolamines (LPEs) contribute to genetic instability and cancer initiation via enhanced phospholipase A2 activity^37^ and inflammation.^3^ Phospholipids are needed in cancer cells to generate the cellular membrane and maintain membrane integrity.^3^ A large metabolic profiling study^3^ of 448 plasma samples from epithelial ovarian cancer patients identified 53 specific metabolites that distinguished early- and late-stage ovarian cancer with an AUC of 0.88. These metabolites included LPCs and LPEs, which were elevated in localised ovarian cancer but reduced in metastasised ovarian cancer. A potential explanation for the reduced levels of LPCs and LPEs in advanced cancer could be that rapidly proliferating tumours consume more phospholipids in their attempt to maintain membrane integrity, leading to an exhaustion of substrates.^30^ Also lysophosphatidic acid (LPA) has been purported to be a possible biomarker because some studies have shown LPA to be elevated in plasma samples of ovarian cancer patients.,^34,38^ but we could not confirm this as we did not monitor LPAs in our lipidomic method.

Borderline ovarian tumours have low malignant potential and elevated mitotic activity without stromal invasion. They commonly occur in younger women compared to ovarian cancer patients and have lower recurrence rates.^39^ Denkert et al.^20^ found significantly different metabolite levels (including metabolites from glycerolipid metabolism and free fatty acids) in borderline ovarian tumour tissues compared to invasive ovarian carcinomas using gas chromatography/time-of-flight mass spectrometry. However, they had only nine borderline tumours in their study. Based on the present study, lipid metabolism in borderline ovarian tumours differs from that in invasive cancers. The Charité cohort had more borderline ovarian tumours (*N* = 18) than the Finnish cohort. These samples were mainly serous epithelial tumours. Significant differences were observed only for occasional plasmalogens as compared to benign controls.

In the Charitè cohort, the results were evaluated by menopausal status. Greater alterations in Cer d16:1, d18:0 and d18:1 were observed in postmenopausal women. However, in some PC lipids, premenopausal changes were stronger. A serum lipidomics study of ovariectomised healthy rats showed that Cers and phospholipids increased in response to oestrogen deficiency while TAGs decreased, which was contrary to earlier studies.^40^ Our study lacks data on possible hormone replacement or hormone therapy in the Charitè premenopausal group. The samples from the Finnish cohort were postmenopausal with no current hormone therapy.

Our study had some limitations. First, changes in lipoprotein levels can at least partly explain the overall decrease of lipids among cancer patients, but unfortunately, we did not have lipoprotein levels available from the patients. Neither did we have the information on body mass index, which may also affect lipid levels. Second, in the Finnish cohort, the sample sets contained both serum and plasma samples, which may affect the lipid levels. However, it is worth noting that the lipid changes were consistent with the two other data sets, and thus, it can be assumed that the difference does not significantly affect the results. Moreover, the logistic regression models developed in the Charité cohort showed high AUC values in the Finnish cohort, which also supports the validity of the results. Third, there was an age imbalance in the cohorts, as the Charité cohort patients were older than the controls. However, the results were consistent with the Finnish cohort, where the controls were older than the patients. This finding and our previous age-adjusted lipidomic analyses^9^ suggest that age does not explain the differences in lipid metabolism observed in ovarian cancer patients. Fourth, the blood samples were not collected during a fasting condition, which may affect the results. However, it is worth noting that there were no differences between groups and that it is expected that fasting samples might have given a better separation between the ovarian cancer patients and the subjects with benign disease.

We have shown that blood lipidomic changes occur in several patient cohorts and already at the early-stage ovarian cancer, but intensify with the progression of the disease. Many of the lipid changes are similar in patients with serous, mucinous and endometrioid ovarian carcinoma, suggesting that rewiring of lipid metabolism is an integral part of ovarian carcinogenesis. The results provide an excellent basis for further development of diagnostics and the future investigations should also explore the potential of exploiting the altered ovarian cancer lipid metabolism for therapeutic purposes.

## Electronic supplementary material

Supplementary Table S1

Supplementary Table S2

## Availability of data and materials

The data sets generated during and/or analysed during the current study are available for non-commercial use from the corresponding author on reasonable request.
